# A meta-analysis of transcriptomic characterization revealed extracellular matrix pathway involved in the H5N1 and H7N9 infections

**DOI:** 10.18632/oncotarget.19315

**Published:** 2017-07-18

**Authors:** Feng Wen, Jinyue Guo, Guangzhi Tong, Dingren Bi, Qi Wang, Xiaomin Liu, Shuaiyong Wang, Tonglin Shan, Wu Tong, Yanjun Zhou, Guoxin Li, Hai Yu

**Affiliations:** ^1^ Division of Swine Infectious Diseases, Shanghai Veterinary Research Institute, Chinese Academy of Agricultural Sciences, Shanghai 200241, China; ^2^ College of Veterinary Medicine, Huazhong Agricultural University, Wuhan 430070, China; ^3^ Jiangsu Co-innovation Center for Prevention and Control of Important Animal Infectious Diseases and Zoonoses, Yangzhou 225009, China

**Keywords:** influenza A, H7N9, H5N1, meta-analysis, extracellular matrix

## Abstract

Avian-origin H5N1 and H7N9 influenza A viruses are capable of causing lethal infection in humans, with serious lung pathology and leading to acute respiratory distress syndrome. The contribution of host response associated with the poor prognosis of H5N1 and H7N9 infections remains unclear. The aim of this study was to identify the host factors involved in the high pathogenicity of H5N1 and H7N9 by a systematical meta-analysis. The RNA-seq datasets related to H5N1, H7N9, and H1N1 infections with time series were retrieved from GEO. After merging the data from different series, ComBat was used to adjust the known variances from different batches. The transcription factors binding the genes in each cluster were predicted by PASTAA. We figured out the genes that were differentially expressed at any time point in samples infected with H5N1, H7N9, or H1N1. The analysis of biological function showed that genes related with cytokine were up-regulated in all three viruses. However, genes associated with carbon metabolism were found exclusively down-regulated in H7N9 and the extracellular matrix pathway were only enriched in H5N1 and H7N9. To summary, our study suggested that the extracellular matrix might be associated with the high fatality of H5N1 and H7N9 viruses in humans.

## INTRODUCTION

Avian influenza, the infections of birds with avian influenza type A viruses (IAVs), occurred naturally among wild aquatic birds worldwide. Avian influenza viruses do not commonly infect humans, but human infections are reported sporadically. Recently, outbreaks of human infections after contact with infected birds or their secretion or through limited person-to-person transmission caused the attention of the public. Following the first appearance of the H5N1 in 1997, the H7N9, H10N8, and H5N6 subtypes of influenza A virus were detected following under the ongoing surveillance efforts, which have all caused severe infections [1].

Among all avian IAVs, H7N9 and H5N1 have been responsible for most human infections worldwide to date, including the most serious illnesses and deaths. Since the first recognized human case of H5N1 infection in 1997, the World Health Organization (WHO) has reported 854 confirmed human infection cases as of July 2016, with a fatality rate of about 66% [1, 2]. Besides, a total of 1307 laboratory-confirmed human infections with the H7N9 has been reported through the notification of the International Health Regulations (IHR) since early 2013 with a fatality rate of 40.4% [1, 3]. More than 400 additional laboratory-confirmed cases of human infection have been reported to WHO from China in the recent months since 2017 [4]. Fortunately, both H5N1 and H7N9 were reported to have limited potential for human-to-human transmission, but it might gain the ability to spread between people through the antigenic shift or antigenic drift. Such risk urged us to prevent and cure the infection of H5N1 and H7N9.

Unlike the common seasonal flu, H5N1 and H7N9 viruses were highly pathogenic. H5N1 was highly pathogenic in both human beings and birds [5], while H7N9 seemed to exhibit low pathogenicity in birds with the severe disease that occurs in human [6]. There was more H7N9 human infections than H5N1, partly because of asymptomatic infection of H7N9 caused by the transmission through direct contact with seemingly healthy but infected birds [7]. FDA approved the first monovalent adjuvanted vaccine for prevention of H5N1 avian influenza in 2013 [8]. This vaccine could be used in the event that the H5N1 avian influenza virus develops the capability of efficient human-human transmission. However, there currently is no publicly available vaccine to protect against H7N9 virus infection [9].

For H5N1 and H7N9, the exact contribution of individual viral effect to pathogenicity at the molecular level is still largely unknown, which is certainly helpful to decrease the fatality. Microarray is a useful tool to understand their infection at the level of transcriptional regulation in the host cells which is important for the interpretation of these complex genetic changes [10]. However, a lot of studies have reported that findings of microarray data were not reproducible or were sensitive to the data perturbations [11, 12]. Moreover, microarray used over 10 thousand probes on tens or hundreds of samples, which exacerbated the accuracy of the potential predictors. As a result, a meta-analysis was used to increase the reliability and generalizability of results.

Through this meta-analysis, we aimed to obtain a more precise set of differentially expressed genes and analyze their biological functions. In this study, we utilized the 6 and 2 available public microarray datasets from Gene Expression Omnibus (GEO) repository [13] for H5N1 and H7N9, respectively, to figure out the genes which were differentially expressed in cell lines infected with influenza virus and control. Another two datasets of H1N1 were analyzed as a comparison, so that we were able to figure out the possible cause of the high pathogenicity of H5N1 and H7N9 and point out the direction to clinical treatment.

## RESULTS

### The data pre-processing for microarray meta-analysis

As the basis for the meta-analysis, there were totally 6 and 2 GEO datasets with time series available for H5N1 and H7N9 respectively and we also selected another 2 datasets from H1N1 to compare the avian influenza with the 2009 pandemic flu in order to figure out why H5N1 and H7N9 were so pathogenic (Table 1).

**Table 1:** The information of the GEO datasets analyzed in this study

Strain^a^	GEO accession	Platform	Submission date	Pubmed ID	Cell Line
H5N1	GSE76599	GPL13497	Jan 06 2016		Calu-3
	GSE66597^b^	GPL6480	Mar 06 2015	26008703	U251
	GSE49840	GPL17077	Aug 13 2013	24496798	Calu-3
	GSE43203	GPL6480	Dec 29 2012		Calu-3
	GSE43204	GPL6480	Dec 29 2012		Calu-3
	GSE28166	GPL6480	Mar 24 2011	21865398	Calu-3
H7N9	GSE49840	GPL17077	Aug 13 2013	24496798	Calu-3
	GSE69026	GPL13497	May 19 2015		Calu-3
H1N1	GSE80697	GPL13497	Apr 26 2016		Calu-3
	GSE40844	GPL6480	Sep 12 2012		Calu-3

^a^ The virus strains selected in this study were: A/Vietnam/1203/2004 (H5N1); A/Anhui/01/2013 (H7N9); A/California/04/2009 (H1N1).

^b^ The dataset annotated was not in the analysis considering it was aberrant after normalization and batch adjustment.

**Figure 1:**
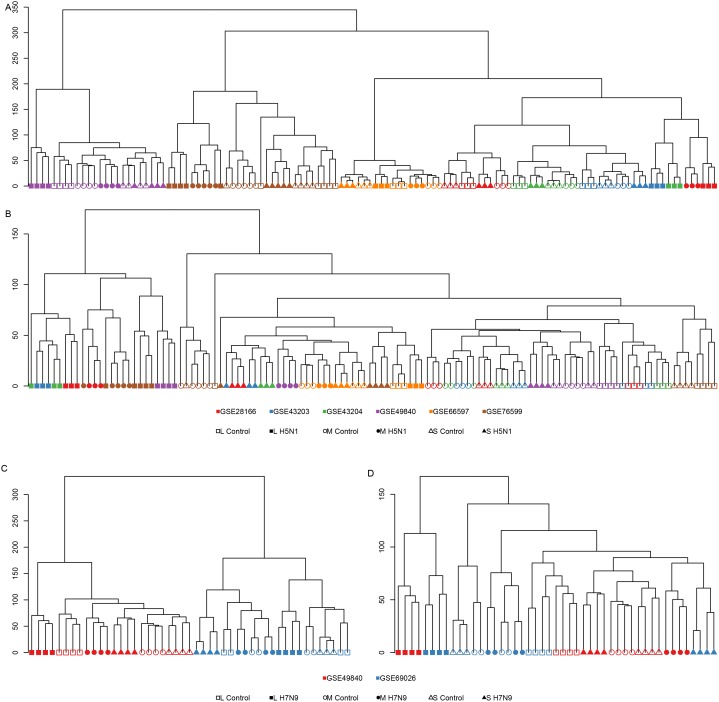
The hierarchical clustering of gene expression levels **(A)** and **(B)** showed the clustering of H5N1 datasets before **(A)** and after **(B)** batch adjustment. **(C)** and **(D)** showed the clustering of H7N9 datasets before and after batch adjustment. The different GEO series were annotated by different colors and different time points were annotated as different shapes. Hollow and solid symbols represented the samples with mock infection and with virus infection.

Because it was generally agreed that microarray data from distinct experimental platforms, often using distinct reference samples, are not directly comparable, we used some strategies to overcome it. After background correction and quantile normalization, the expression level of each gene was estimated by the median of the expression levels of all the probes mapped to it. Next, ComBat was utilized to adjust the known dataset differences with an empirical Bayesian framework. The hierarchical clustering of the expression profiles before and after adjustment indicated that our strategies worked for meta-analysis. Before adjustment, the samples from the same datasets were clustered together (Figure 1A, 1C). But after adjustment, the samples from the same time points were the closest to each other (Figure 1B, 1D) with an exception for dataset GEO66597 and the 7h time point. Considering its aberrant clustering performance and the fact that the cell line used in GEO66597 were different from other studies, which meant that they used different tissues as a target, we decided to remove it from the following studies. The 12h and 24h time points of H5N1 were clustered together well (Figure 1B), but only 24h time point of H7N9 were clustered together. This suggested two possibilities: first, the limited datasets for H7N9 constrained the performance; and second, the cells might need more time to react when infected with H7N9 than H5N1.

**Figure 2:**
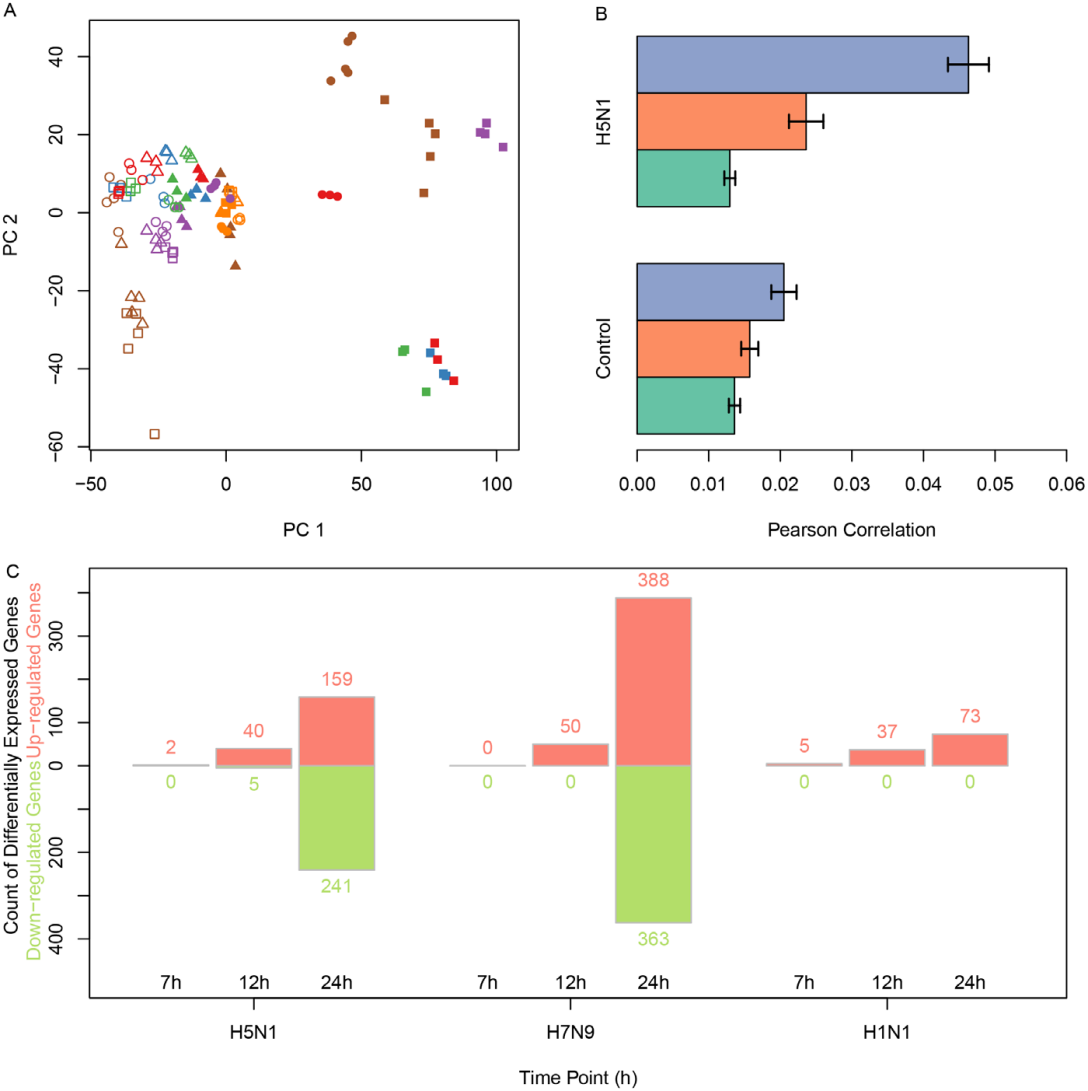
The correlations between H5N1 expression levels and the differentially expressed genes in infected samples **(A)** The principal component analysis of H5N1 datasets showed that samples with H5N1 infections after 12h or 24h were specific to other samples. **(B)** The average distances between H5N1-infected samples and time-matched infected or mock samples. The x-axis was the Pearson correlation distance and error bars indicated standard deviation. **(C)** The count of genes which were up-regulated or down-regulated in H5N1, H7N9 and H1N1 using red and green, respectively.

The principal component analysis also showed the time points were the largest possible variance for H5N1 where H7N9 were not shown but had similar results as H5N1 (Figure 2A).

### Identification of differentially expressed genes using microarray meta-analysis

First, we calculated the Pearson correlation of each time point for H5N1 and H7N9. As expected, the internal variance increased as the time passed (Figure 2B, H7N9 not shown). The differentially expressed genes were identified for each time point of H5N1, H7N9, and H1N1. The count of genes significantly up-regulated or down-regulated in every group were plotted as Figure 2C.

**Figure 3:**
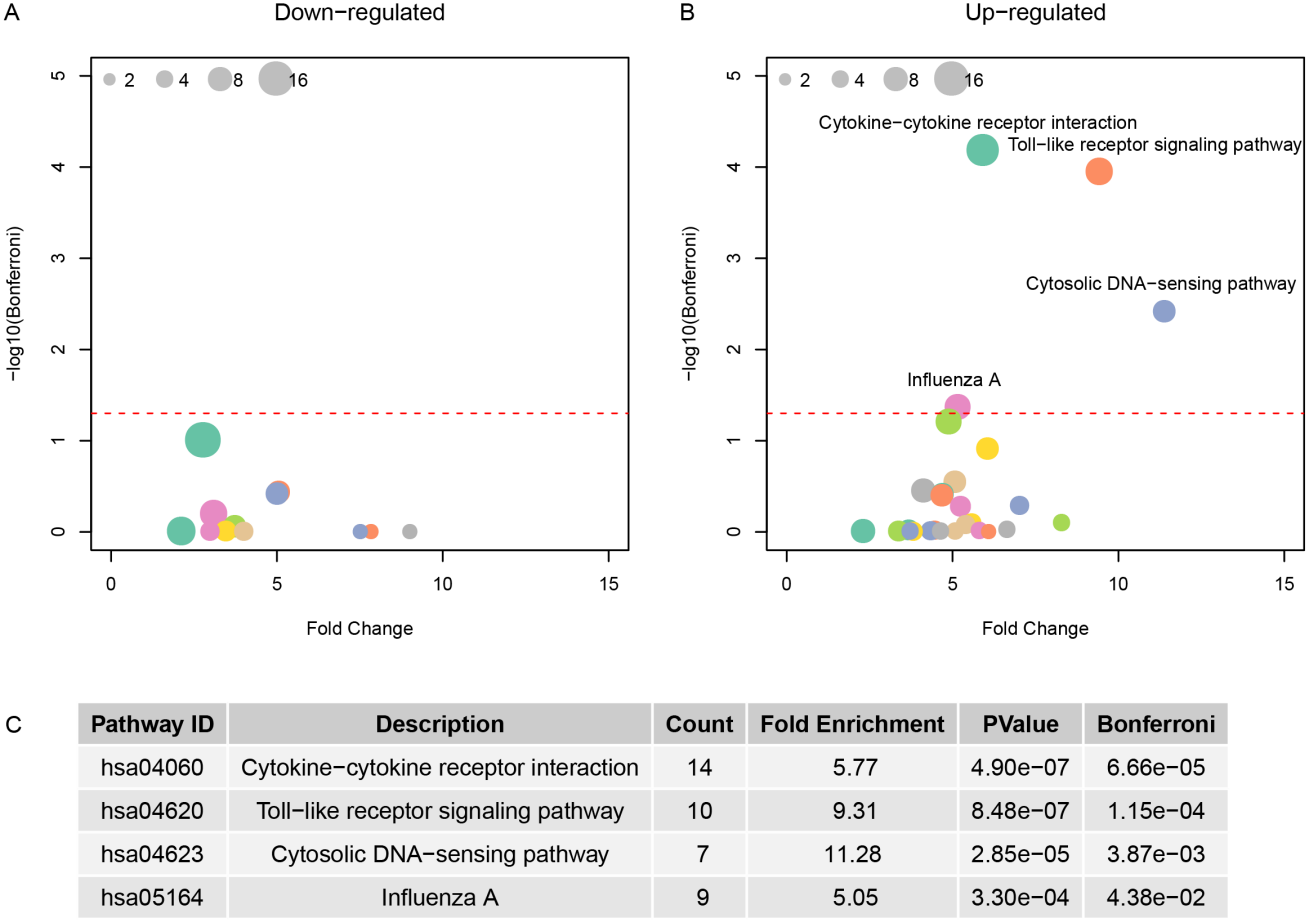
The pathway enrichment for differentially expressed genes in H5N1 **(A)** and **(B)** showed the fold change and negative logarithm of Bonferroni as x-axis and y-axis. And the size of point indicated the genes involved in this pathway. **(C)** The details of significant pathways.

The KEGG pathways were enriched for the up-regulated or down-regulated genes in H5N1, H7N9, and H1N1 virus strain. Figure 3 showed the enriched pathways for H5N1 and the pathways enriched for H7N9 and H1N1 were available in Table 2. The Cytokine-cytokine receptor interaction, Toll-like receptor signaling pathway, Cytosolic DNA-sensing pathway and Influenza A pathway were all up-regulated in cells infected with three virus strains. These results suggested that the cells were using chemokine and cytokines to defense the virus. But the down-regulated genes of the three viruses had different patterns. There were no down-regulated genes available for H1N1, and no enriched pathway for H5N1, while the down-regulated genes in H7N9 were associated with metabolism.

**Table 2:** Enriched pathways for up and down-regulated genes

Category	Term	Count	P Value	Genes	List total	Pop hits	Fold enrichment	Bonferroni	FDR
h7n9 up									
KEGG_PATHWAY	hsa01100:Metabolic pathways	63	2.63E-08	LDHA, ALG1, HEXB, HLCS, ALG8, AUH, FDFT1, AKR1C3, MTHFD1, PIGK, IDH3G, RPN1, SUCLA2, DHCR24, PTDSS2, PLD1, ACO1, ALDH5A1, PFKP, NADSYN1, DPAGT1, PIGT, PFKM, GMPS, PIGO, GNS, MAN2A1, PLCE1, PGM1, RRM1, PCCB, EXT2, ALDOA, GALNT2, NAGLU, GANAB, CYP51A1, POLA2, HADHA, ISYNA1, STT3A, DHCR7, PEMT, IDH2, FASN, B3GNT3, ACSL4, PAPSS2, GBA, ENO1, FH, DGKQ, MAOB, TKT, ACSM3, MPI, MTR, PHGDH, ALDH2, ALOX5, PGK1, CBS, PYGB	170	1158	1.964309662	5.93E-06	3.36E-05
KEGG_PATHWAY	hsa01130:Biosynthesis of antibiotics	22	1.08E-07	ALDOA, LDHA, ACO1, CYP51A1, PFKP, TKT, PFKM, HADHA, FDFT1, ISYNA1, IDH3G, PGM1, PHGDH, ALDH2, IDH2, PGK1, SUCLA2, PAPSS2, PCCB, CBS, ENO1, FH	170	201	3.951887621	2.45E-05	1.38E-04
KEGG_PATHWAY	hsa01230:Biosynthesis of amino acids	12	2.27E-06	ALDOA, IDH3G, ACO1, MTR, PHGDH, IDH2, PFKP, TKT, PFKM, PGK1, CBS, ENO1	170	69	6.279283887	5.14E-04	0.00290813
KEGG_PATHWAY	hsa01200:Carbon metabolism	14	6.98E-06	ALDOA, ACO1, PFKP, TKT, PFKM, HADHA, IDH3G, PHGDH, IDH2, PGK1, SUCLA2, PCCB, FH, ENO1	170	108	4.680392157	0.0015757	0.0089203
h7n9 down									
KEGG_PATHWAY	hsa05168:Herpes simplex infection	15	1.49E-06	IL6, TNF, SP100, CREBBP, NFKBIA, OAS1, OAS2, CFP, IFIT1, IFNB1, IRF7, JUN, IFNA4, SRSF8, IFNA8	110	170	4.923529412	2.53E-04	0.00181355
KEGG_PATHWAY	hsa05164:Influenza A	14	4.52E-06	IL6, TNF, CREBBP, NFKBIA, OAS1, OAS2, CXCL10, IFNB1, TNFRSF10D, IRF7, JUN, IFNA4, IFNA8, MX1	110	161	4.852173913	7.69E-04	0.0055202
KEGG_PATHWAY	hsa04620:Toll-like receptor signaling pathway	11	7.12E-06	IL6, TNF, LY96, IFNB1, JUN, IRF7, IFNA4, NFKBIA, IFNA8, CD14, CXCL10	110	97	6.327835052	0.00121	0.00868811
KEGG_PATHWAY	hsa05162:Measles	12	1.33E-05	IL6, TNFRSF10D, IFNB1, IRF7, IFNA4, NFKBIA, IL13, OAS1, OAS2, IFNA8, MX1, TNFAIP3	110	127	5.272440945	0.0022503	0.01616612
KEGG_PATHWAY	hsa04622:RIG-I-like receptor signaling pathway	9	1.66E-05	TNF, ISG15, IFNB1, IRF7, IFNA4, NFKBIA, IFNA8, DHX58, CXCL10	110	65	7.726153846	0.0028259	0.02030615
KEGG_PATHWAY	hsa04060:Cytokine-cytokine receptor interaction	15	2.83E-05	CSF2, IL6, TNF, PDGFA, IL13, IL11, CXCL10, CCR7, IL23A, IL20RB, IFNB1, IFNA4, CX3CR1, IFNA8, LTB	110	219	3.821917808	0.0048037	0.03455014
KEGG_PATHWAY	hsa04623:Cytosolic DNA-sensing pathway	8	5.20E-05	IL6, IFNB1, IRF7, IFNA4, NFKBIA, IFNA8, CXCL10, ZBP1	110	56	7.971428571	0.0088	0.06341132
h1n1 down									
KEGG_PATHWAY	hsa05164:Influenza A	12	3.38E-10	IL6, IFIH1, IRF7, OAS3, RSAD2, OAS1, OAS2, CCL5, MX1, STAT1, IL1A, CXCL10	34	161	13.45560833	2.64E-08	3.58E-07
KEGG_PATHWAY	hsa05168:Herpes simplex infection	10	1.69E-07	IFIT1, IL6, IFIH1, IRF7, TAP1, OAS3, OAS1, OAS2, CCL5, STAT1	34	170	10.61937716	1.32E-05	1.79E-04
KEGG_PATHWAY	hsa05162:Measles	9	2.42E-07	IL6, IFIH1, IRF7, OAS3, OAS1, OAS2, MX1, STAT1, IL1A	34	127	12.79342288	1.89E-05	2.56E-04
KEGG_PATHWAY	hsa04620:Toll-like receptor signaling pathway	6	1.49E-04	IL6, IRF7, CCL5, CXCL11, STAT1, CXCL10	34	97	11.16676774	0.0115197	0.15700528
KEGG_PATHWAY	hsa04623:Cytosolic DNA-sensing pathway	5	2.09E-04	IL6, IRF7, CCL5, ZBP1, CXCL10	34	56	16.11869748	0.0161644	0.22075685
KEGG_PATHWAY	hsa04668:TNF signaling pathway	6	2.26E-04	CXCL1, CSF2, IL6, CXCL2, CCL5, CXCL10	34	106	10.21864595	0.0174505	0.2384561
KEGG_PATHWAY	hsa04062:Chemokine signaling pathway	7	3.35E-04	CXCL1, CCR7, CXCL2, CCL5, CXCL11, STAT1, CXCL10	34	180	7.020588235	0.0258013	0.35386457
KEGG_PATHWAY	hsa05160:Hepatitis C	6	4.18E-04	IFIT1, IRF7, OAS3, OAS1, OAS2, STAT1	34	121	8.951871658	0.0320867	0.44129508

### Identification of clusters of expression profiles in virus strains

**Figure 4:**
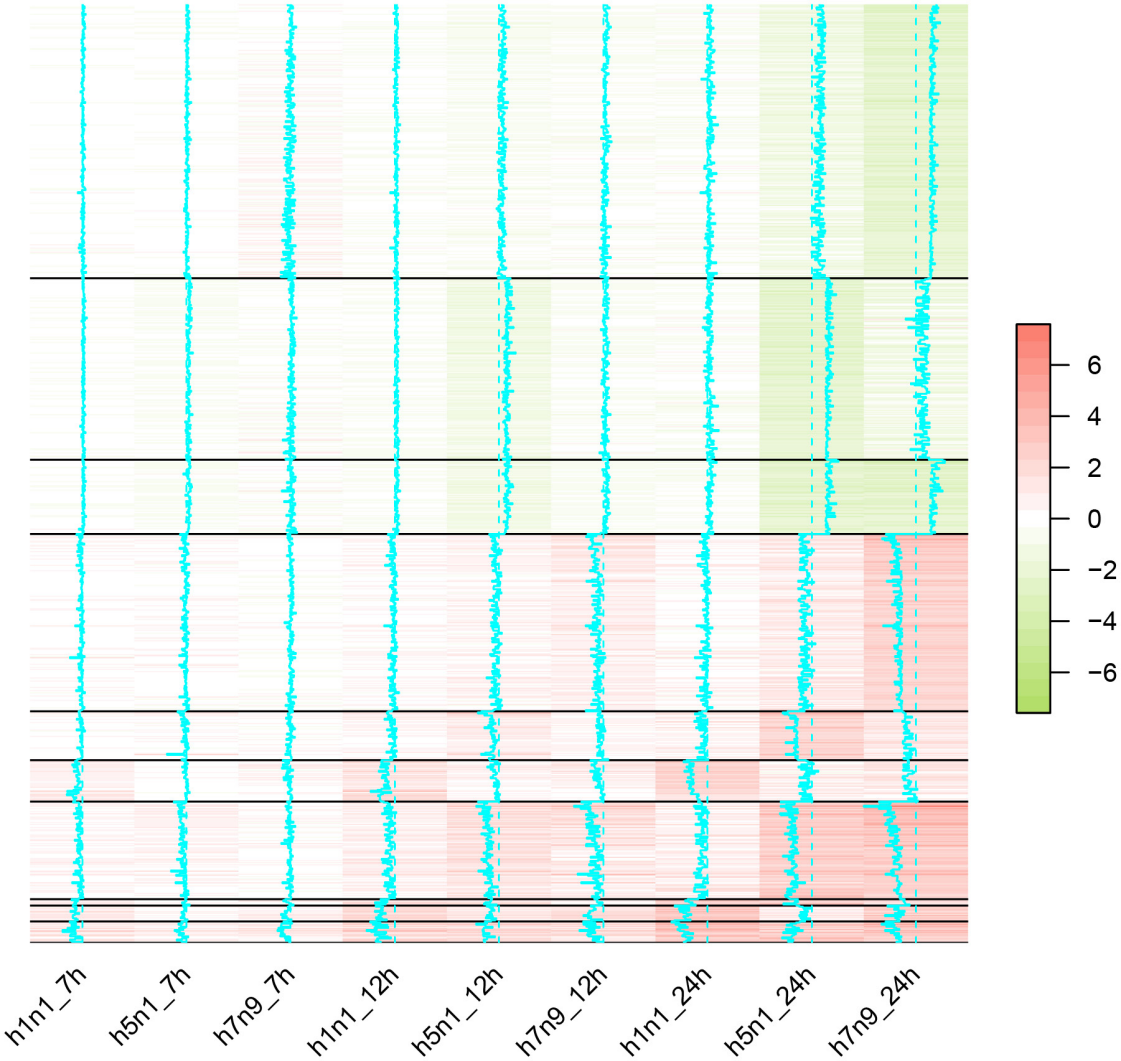
The heatmap of differentially expressed genes in H1N1, H5N1 or H7N9 datasets Each column represented one virus strain at a time point and each row represented one differentially expressed genes. The distance of the trace line from the center of each color-cell is proportional to the size of the measurement. Different clustering of genes were separated by the solid black lines.

The fold changes of genes that were differentially expressed in any strain were utilized to decide the clusters of the expression profiles (Figure 4). The expression levels of most genes were only changed dramatically in 24h time point. Moreover, the correlation between H5N1 and H7N9 at 24h time point (Pearson correlation: 0.82) were higher than the correlation between H5N1 and H1N1 (Pearson correlation: 0.61) or H7N9 and H1N1(Pearson correlation: 0.59). It suggested that H5N1 and H7N9, both of which were avian influenza viruses, were more similar to each other.

Finally, we obtained 10 clusters based on their expression profiles in H5N1, H7N9, and H1N1. Based on the genes involved in 10 clusters, the involved pathways and the enriched transcript factors were summarized in Table 3. Some clusters did not have enriched pathway or transcript factors, which might by led from a few genes involved. The only pathways enriched for up-regulated clusters were Influenza A pathways and most of the transcript factors belonged to interferon regulatory transcription factor (IRF) family. It suggested that the up-regulated genes were highly associated with the interferon regulation and were common in both avian influenza and pandemic flu. Besides, Sta5a and Sta5b mediate cellular responses to the cytokine KITLG/SCF and other growth factors, which might help to fight against infection.

**Table 3:** The biological function analysis of genes in each cluster generated by fold change of expression levels in virus-infected samples and controls

Cluste^a^	Gene	Attributes	Enriched pathway	TF^b^
1	258	down-regulated in H7N9	Metabolic pathways	Pax-5 Sp1 Egr-1 Egr-2 Hif-1α Ap-2γ
			Biosynthesis of antibiotics	
			Carbon metabolism	
			Biosynthesis of amino acids	
			Glycolysis/Gluconeogenesis	
			N-Glycan biosynthesis	
			Protein processing in endoplasmic reticulum	
2	171	down-regulated in H5N1	NA	Creb
				Crebβ
3	70	down-regulated in H5N1 and H7N9	ECM-receptor interaction	NA
4	167	up-regulated in H7N9	NA	NA
5	46	up-regulated in H5N1	NA	NA
6	39	up-regulated in H1N1	Influenza A	Irf-7a Irf-1 Irf-10 Irf-8 Stat5a Stat5b
7	6	up-regulated in H1N1 and H5N1	NA	NA
8	15	up-regulated in H1N1 and H7N9	Influenza A	Irf-1 Irf-10
9	92	up-regulated in H5N1 and H7N9	NA	NA
10	20	up-regulated in all	NA	Irf-1 Irf-10 Irf-7a Irf-2 Irf-8

^a^ The adjusted p-values by Benjamini method of enriched pathway was lower than 0.05.

^b^ TF: transcriptional factors, the association scores of PASTAA was larger than six.

However, the down-regulated clusters had different performance. First, cells infected H1N1 viruses did not have any down-regulated genes in this study surprised but it might be caused by the strict criteria for differentially expressed genes and the large amount of up-regulated genes. Second, the genes only down-regulated in H7N9 were highly associated with metabolic pathways, while the genes down-regulated in both H7N9 and H5N1 were related to the extracellular matrix pathway. Though there was no pathway enriched for genes only low-expressed in H5N1, the transcript factor Creb and Crebβ might have an impact.

## DISCUSSION

Influenza viruses can be categorized as either low pathogenicity or high pathogenicity based on their ability to induce disease in a specific host. A better understanding of mechanisms that may lead to severe illness or death caused by high pathogenicity H5N1 or H7N9 would benefit the treatment of those infections when happens in humans. Studies of differences in the host response to different virus strains would provide insight into why viruses exhibit severe damage to then certain host. In this study, we preformed a meta-analysis to evaluate the host response to two high pathogenicity H5N1 and H7N9 avain influenza viruses with the comparison with the seasonal flu H1N1, which have been reported to cause high mortality in human infection cases.

As describe in the results, cells infected with H5N1, H7N9, and H1N1 shared the similar functions of up-regulated genes. They were the cytokine-cytokine receptor interaction, Toll-like receptor signaling pathway, cytosolic DNA-sensing pathway and influenza A pathway. The transcription factors were also similar which were associated with the interferon regulatory transcription factor (IRF) family. As expected, most of the up-regulated genes were associated with the immune system against infection. However, the down-regulated genes showed various patterns for H5N1 and H7N9, while there were no down-regulated genes in H1N1.

We failed in obtaining the enriched pathway for the genes that were only down-regulated in H5N1. In order to understand their function in another way, we also utilized STRING v10 as an alternative to look at their network status and GO enrichment [14]. It showed that these genes were significantly related with positive regulation of cell migration and negative regulation of cell differentiation. And also these genes seemed to have significantly more interactions than expected, which indicated that the genes were at least partially biologically connected as a group. As a result, we could conclude that the low-expression of these genes would increase cell differentiation and decrease cell migration, which in further might be related with virus-elicited inflammatory and immune reactions.

The cluster with genes only down-regulated in H7N9 was associated with metabolic pathways, especially the carbon metabolism. Qin, Zhang [15] also reported that H7N9 infection could be linked to saccharide or polysaccharide metabolism. It also reported that central metabolism could be strongly affected by virus infections [16]. Besides, biosynthesis of amino acid was also enriched. Thus, the carbon synthesis and amino acid synthesis might be essential to the virus replication of H7N9.

Finally, the down-regulated genes shared by H5N1 and H7N9 were related with the extracellular matrix. The KEGG pathway, the extracellular matrix (ECM), was defined as a complex mixture of structural and functional macromolecules and serving an important role in tissue and organ morphogenesis and in the maintenance of cell and tissue structure and function [17]. The functional macromolecules in the extracellular matrix contained proteoglycans, non-proteoglycan polysaccharide, collagens, fibronectin and laminin and so forth. Several studies have reported the relationship between ECM and viral infections. For example, ECM and interacting proteins involved in the entry of various viruses like gamma-retrovirus, hepatitis B virus and rhabdovirus [18, 19]. Leung, Li [20] reported that treating cells with anti-fibronectin antibodies or fibronectin-specific small interfering RNA inhibited the H1N1 replication, but did not inhibit the H5N1 viruses. Moreover, H3N2 virus was able to use intercellular connections to spread to neighboring cells [21]. These reports indicated that H5N1 and H1N1 replicated with the help of the extracellular matrix in a different manner and the extracellular matrix helped the virus entry, replication, and spread. And according to the functional analysis, H7N9 should be similar to H5N1 in this aspect. Additionally, Chen, Zhou [22] also reported that EMC pathway was involved in H7N9 infection. On the other hand, Chen, Cui [23] found that higher plasma levels of hydrolysis of fibronectin and collagens IV related proteins in survivors of severe H7N9 infection and they hinted the ongoing tissue remodeling after severe H7N9 infection. Thus, the perturbed extracellular matrix pathway in cells might also hint the damaged tissue remodeling of cells, which devastated the fatality.

To summary, we performed a systematic meta-analysis to evaluate the expression profiles in samples infected with H5N1 and H7N9 and we suggested that the extracellular matrix worked positively in the high fatality of H5N1 and H7N9 through affecting viral replication and spread and reducing tissue modeling.

## MATERIALS AND METHODS

### Data selection and characteristics

“H5N1”, “H7N9”, and “H1N1” were used, respectively, as keyword when search GEO series (https://www.ncbi.nlm.nih.gov/geo/). The *Homo Sapiens* and expression profiling by array were used as filtering for the organism and series type. A total of 9 series were available for H5N1, among which 6 series (GSE76599, GSE66597 [24], GSE49840 [25], GSE43203, GSE43204, and GSE28166 [26]) had time series data and were included in this study. Only 2 series (GSE49840 [25] and GSE69026) of H7N9 datasets met our criteria. To compare the host response of highly pathogenic avian influenza with the pandemic H1N1 flu, 2 series of H1N1datasets (GSE80697 and GSE40844) were randomly selected using the same criteria. The virus strains selected in this study were: A/Vietnam/1203/2004 (H5N1); A/Anhui/01/2013 (H7N9); A/California/04/2009 (H1N1). The detail characteristics of the GEO data series were listed in Table 1. All the datasets were generated with the Agilent platform. To prevent the differences introduced by different methods used in data preprocessing of these series, we downloaded the raw data and processed them using the same procedures. The R package GEOquery [27] was utilized to download the raw Cel files and also the information of each sample. The platform information of every series was retrieved directly from the GEO website.

### Microarray data pre-processing

The data pre-procession were carried out with the help of the limma package in R software [28]. All the included microarray data used single-channel Agilent platform, so only the intensities of the green channel were extracted. The medians of the foreground and background pixels were calculated as the estimated foreground and background signals. Next, we adjusted the foreground adaptively for the background intensities based on convolution model using the method called ‘normexp’, which was preferable to background subtraction when assessing differential expressions [29]. Finally, quantile-normalization was applied to the data. Only the mock-infected samples and the samples infected with the targeted influenza were kept into the next step.

### Data merge and batch adjustment

The gene names were mapped to the probes based on the platform information. The expression level of each gene was calculated using the median value of the probes that were mapped to it. The data from different series were merged together according to the gene symbol and any genes missing in any series were excluded. The batch adjustment between different GEO series was completed using package SVA in R software [30]. ComBat function was effective to adjust for known batches using an empirical Bayesian framework [31].

In order to confirm the effects of batch adjustment and also the prerequisite of this meta-analysis study, the expression levels before and after adjustment were visualized using hierarchy clustering and principal component analysis. One series (GSE66597) seemed to be an outlier and was removed in the following study.

### Differentially expressed genes identification

The differentially expressed genes on each time point of all three H5N1, H7N9, and H1N1 were identified through limma package by comparing the samples with virus infections and with mock infection [28, 32]. Linear models were fitted and empirical Bayes method was used for statistical analysis and assessing differential expression. The obtained p-values were adjusted by false discovery rate (FDR). The genes with FDR less than 0.01 and absolute log2-fold-change larger than 2 were considered as significantly differentially expressed genes.

The genes that were differentially expressed in any virus strain were extracted and they were clustered based on their logarithm of fold change on three time points in different strains and 10 different clusters were found.

### Biological function analysis

The KEGG pathways were enriched using DAVID Bioinformatics Resources v6.8 [33, 34] for genes up-regulated or down-regulated in samples infected with any influenza and for genes belonging to different clusters. HumanGenome in Agilent Backgrounds was selected as population background, considering that all the datasets used Agilent platform. For genes up-regulated or down-regulated in H5N1, H7N9 or H1N1, the p-values were adjusted using Bonferroni methods. However, due to the limited genes in each cluster, the p-values of enriched pathways for different clusters were adjusted using Benjamini method, which is looser than Bonferroni.

The transcription factors binding the genes in each cluster were predicted by PASTAA [35]. Only the transcription factors with an association score larger than 6 were considered as an enriched transcription factor for each cluster.
